# Multispecialty sessions model for comprehensive care and decision-making in cancer patients

**DOI:** 10.3332/ecancer.2025.1830

**Published:** 2025-01-22

**Authors:** Kiara S Berumen, Alberto Sánchez-Navarro, Andrea Velázquez, Manuel Solano, Francisco Anaya, Gustavo Rosales, Alexandra Díaz, Johana Jazer, Kathia Zamudio, Susana Suder, Marisol Quintero, Adriana González-Martínez, Luis A García

**Affiliations:** 1 School of Medicine, Universidad del Valle de Atemajac, Guadalajara 45050, Jalisco, México; 2 Instituto Oncológico Nacional, Guadalajara 44600, Jalisco, México; 3 School of Medicine, Universidad Autónoma de Guadalajara, Guadalajara 45129, Jalisco, México; a https://orcid.org/0009-0005-0348-5538; b https://orcid.org/0000-0003-0697-9136; c https://orcid.org/0000-0003-0358-9167; d https://orcid.org/0000-0001-9059-1892; e https://orcid.org/0009-0002-0767-3702; f http://orcid.org/0000-0002-8275-8004; g https://orcid.org/0000-0003-3282-7531; h https://orcid.org/0009-0007-9411-7478; i https://orcid.org/0009-0007-0070-5886; j https://orcid.org/0000-0002-7503-2141; k https://orcid.org/0009-0003-4471-8962; l https://orcid.org/0000-0001-7221-2377; m https://orcid.org/0000-0001-9395-7087; † These two authors also contributed to this document.

**Keywords:** oncology patient, multispecialty sessions, comprehensive approach, specialty units, decision making, outcome optimisation, health care teams, outcome evaluation, specialist collaboration, virtual sessions

## Abstract

The management of the oncology patient is complex, due to late diagnosis, treatment and costs, affecting the medical, psychological, nutritional and economic areas. The creation of a multispecialty team, with specialists from health and other fields, is the characteristic of the institute. Since 1920, multidisciplinary has evolved and stood out in developed countries; in Mexico and continues to grow. Although it presents challenges, such as lack of time and coordination, its implementation significantly improves the comprehensive approach to patients.

**Methods:**

A retrospective study conducted at the Instituto Oncológico Nacional (ION) in Guadalajara, Mexico, was conducted from April 2019 to December 2023, involving multispecialty sessions via Zoom for oncology patients. Information were collected from medical records with specific inclusion criteria, for the creation of a database in Excel and subsequent analysis with the IBM SPSS Statistics 22 tool.

**Results:**

93.09% of the patients were placed in a specialised unit, while 6.91% in ‘other tumours’. 37.2% of the sessions were held to establish treatment, 32% for diagnosis and 30.8% for both. The breast tumour unit had the most sessions (15%) and the robotic surgery unit had the least sessions (0.8%). 74.9% of the cases required one session and 25.1% required more sessions. An average saving of 5 consultations were generated; 68.8% of the sessions met their objectives and were followed up.

**Conclusion:**

Multispecialty virtual sessions in ION generate significant benefits: they reduce waiting times, save resources, improve access to specialised units and a comprehensive approach. Success is due to team coordination, communication between specialists and patients and implementation of decision making. It is recommended to promote this approach to optimise clinical outcomes and quality of life, overcoming logistical barriers and evaluating its long-term impact.

## Introduction

The management of an oncologic patient is extremely complex, due to the pathophysiology of the disease itself, the late diagnosis, the treatment and the cost involved in the comprehensive approach. This can have a strong impact on several areas of importance for the patient, ranging from the economic and public health services, to the involvement of areas such as nutrition and psychology, which can be impaired both in the short and long term. Therefore, the creation of a multispecialty team that includes a large number of specialists, not only from the health field (medical, nutritional, psychological and nursing), but also from the social, administrative, financial and pharmaceutical fields, involves the alliance between highly trained professionals, guaranteeing individualised management, providing consensual solutions that give confidence and certainty, optimising time, resources and generating strategies with a positive effect on the treatment, survival and quality of life of the oncology patient [1].

The rise of multidisciplinary units and teams made up of medical specialists began more than 5 decades ago [2, 3]. In 1920, Dr. Milligan and his colleagues were already discussing patient cases within the field of laryngology and oncology in a multidisciplinary setting. The authors documented the clinical history of each patient, and the opinions of all the specialists involved and produced a report detailing the treatment applied and the evolution of the patient within a few months [3]. O'Brien [4] made important contributions by recounting his work at Baylor Hospital in the late 1960s and early 1970s; a weekly meeting was held there where medical oncologists, radiation therapists and surgeons discussed various cancer cases together.

Consolidation and expansion took place during the 1990s, where the multidisciplinary approach was adopted in European clinical practice, especially in countries such as the United Kingdom and Germany [5, 6]. In the United Kingdom, during 1996, the Department of Public Health reported its findings in relation to breast cancer, being the first to establish national standards for cancer management from a wide range of evidence-based publications [7]. Similar guidelines were developed for colorectal, lung and gynaecologic cancers, where a multidisciplinary team consensus management represented a consistent recommendation [8]. In other countries, the implementation of this approach occurred later, as is the case in Belgium, where it was established as mandatory in 2000 [6].

Currently, the multidisciplinary resource is used in Mexico in different oncology units of the public health system, such as the National Cancer Institute, the Institute of Social Security of the State of Mexico and Municipalities, the Institute of Security and Social Services for State Workers and the National Medical Center, specifically in the head and neck area. Its specific objectives are to guarantee personalised diagnosis and treatment with the participation of the main oncological areas (oncological surgery, medical oncology and radiation oncology); if necessary, alternate specialties are invited, thus optimising communication, time and agreements through weekly or monthly committee meetings, depending on the complexity [9].

On the other hand, a group of specialists dedicated to the approach to colorectal cancer use another methodology that consists of the prior elaboration of specific questions for each of the selected areas, while at the same time creating an initial basic document as a working guide that is sent electronically to the group. At the time of the meeting, the document is reviewed and the recommendations of each member are given to conclude the best diagnosis or treatment for the patient [10].

At the same time, in regional hospitals in Yucatan, a series of problems have been identified when working with this modality, the main ones being lack of time or disinterest on the part of some physicians, lack of physical space for meetings and the extension of the session time due to deviations from the subject [11]. In spite of this, this practice is considered relevant since it provides the patient with a unique and personalised treatment that improves the course of action and prognosis [12].

On the other hand, a single specialist cannot encompass all of the knowledge and experience needed to create a multi-specialty team that not only involves medical personnel but has improved as a result of this.

For the implementation of sessions with a group of specialists, the main problem is the lack of communication and coordination between them when the patient is treated without a joint evaluation; this can lead to a waste of resources, duplication of diagnostic and therapeutic tests and delay in the approach, thus having a negative impact on the patient's prognosis and follow-up.

Without the participation of different specialties in each individual case, the plan and follow-up may not be the most appropriate for the specific needs of the patient, which are not only biological, but also psychological and financial. On the other hand, the quality of the teams' decision-making depends on their performance, organisation, logistics, information presented and infrastructure for meetings [13].

Therefore, this article aims to evaluate the effectiveness of the multispecialty session model in decision-making regarding the diagnosis and treatment of oncology patients in a private center in Mexico, analysing the capacity to assign patients to specialised units, the relationship between the number of specialties involved and the reduction of consultations, as well as the fulfillment of the objectives in the session and the barriers to follow-up, to optimise the comprehensive approach to these patients.

## Methodology

This study was designed as a retrospective, cross-sectional, non-randomised study with a population determined by convenience. The research was conducted at the Instituto Oncológico Nacional (ION), a private institution located in Guadalajara, Jalisco, Mexico, from April 2019 to December 2023, with the objective of analysing existing data from the multidisciplinary session, regarding diagnosis and decision making.

The study was performed with an institutional request and authorisation for the collection and use of data for research. Since this was a retrospective study, the confidentiality of patient data was guaranteed and the use of personally identifiable information was avoided.

Study participants had to meet the following requirements: be a patient of the institute, have an oncologic diagnosis, have been discussed in the multispecialty virtual sessions during the established time, have a complete medical record and have signed their informed consent.

**Figure 1.**
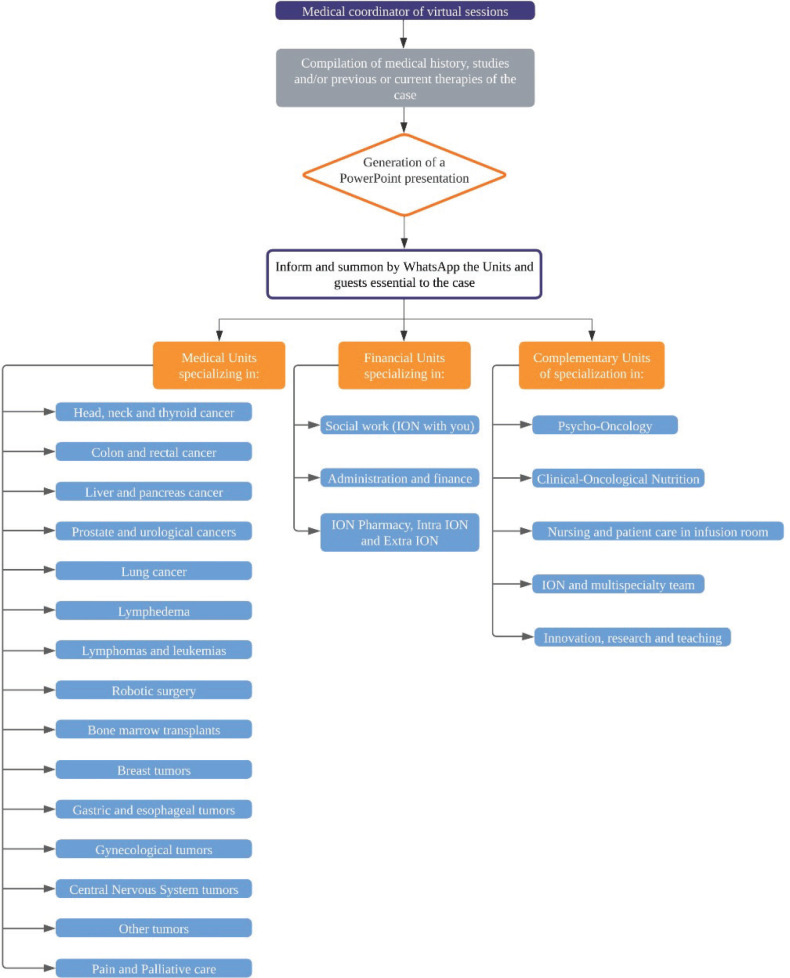
Model of comprehensive oncology patient care based on multispecialty sessions in ION.

Figure 1 shows the methodology used at the National Oncology Institute to provide comprehensive care to oncology patients in a Zoom session on Mondays and Thursdays of each week, specifically at 8:15 a.m., with a maximum duration of 1 hour [14]. It begins with the participation of the leading specialist of the case, who proposes to a coordinating physician the reason behind the multispecialty virtual session. The coordinator is in charge of compiling the case's clinical history, studies (laboratory and cabinet) and/or previous or current therapies. He then creates a PowerPoint presentation to inform and convene the necessary units (medical, financial and complementary) and external guests, so that the specific team for each case is generated and all previous patients have the relevant patient information at their disposal [10]. During the meeting, the purpose of the session is discussed and each team member presents his or her suggestions to the physician in charge of the case; during the meeting, written minutes are generated in Microsoft Word, which are shared digitally with the entire multispecialty team, physically delivered and explained by the treating physician to the patient, so that he or she can make use of the information without restrictions.

**Figure 2.**
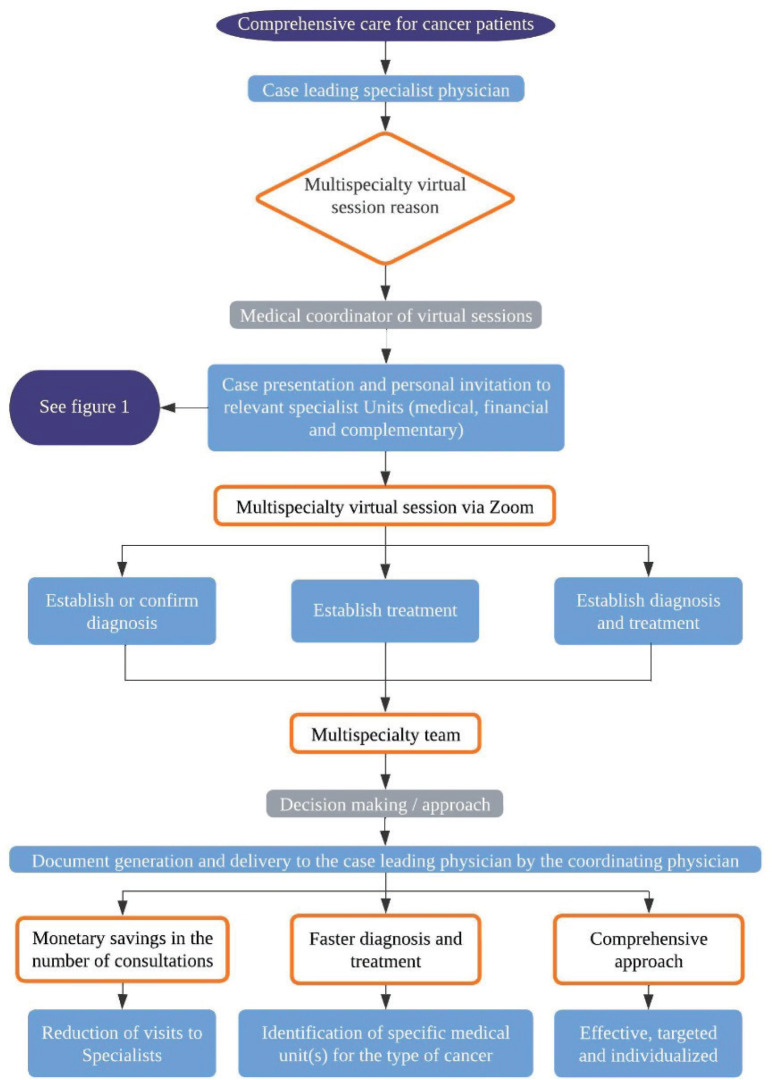
Specialisation units that make up the patient care model in ION.

ION has 15 medical specialty units, as well as complementary units for research, psycho-oncology, clinical-oncology nutrition and nursing. In addition, there are administrative, social work, pharmacy and finance units (Figure 2), which in turn participate and make up the ION team unit in the multispecialty sessions.

An Excel database was created to process the information, using the clinical records to add sociodemographic data of the patients, the reason for the session (to define a diagnosis, a treatment or both), the number of participants in the multispecialty session, the number of sessions, the number of consultations saved and to determine whether the objective of the session was achieved. Descriptive analyses were performed; Spearman’s correlation analysis for the number of specialties/consultations were saved and the number of physicians/consultations were saved; and ANOVA test for the reason for the session and objectives was achieved. All this was done using the IBM SPSS Statistics 22 program.

## Results

**Graph 1.**
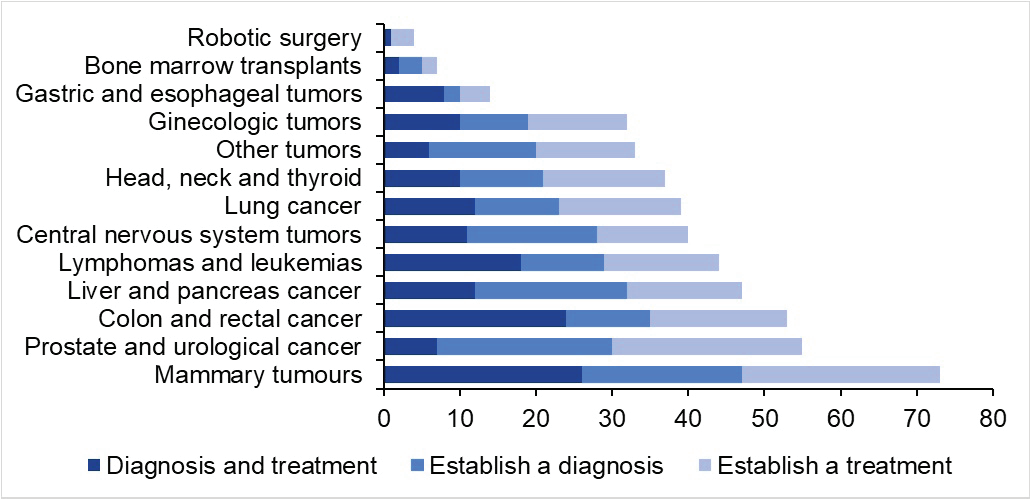
Number of sessions requested and reason for request by ION specialisation units.

We reviewed 590 files of patients whose cases underwent multispecialty sessions, of which 478 met the requirements to be included. 93.09% of the patients could be placed in a specialised commissioned unit according to their cancer type. The 6.91% were non-specific (‘other tumours’), it should be noted that this percentage of patients did not have continuity with their attendance to the institute; therefore, it was not possible to conclude a diagnosis. It is worth mentioning that all the units have requested clinical case sessions. 37.2% of the session requests were to establish treatment, 32% to establish diagnosis and 30.8% to establish diagnosis and treatment. The breast tumour unit has requested more sessions with 15% (*n* = 73), while the robotic surgery unit has requested fewer sessions with 0.8% (*n* = 4) (Graph 1).

There were two clinical cases that were urgently scheduled, due to the type of case, within 24 hours for decision making. There were 11 clinical cases that required more waiting time due to the number of studies required and to have the complete file; this was mainly in the lung cancer, central nervous system tumours, lymphomas and leukemia units. Therefore, the waiting time for a session depends on the type of case and on having all the information to make an individualised and evidence-based decision. The average waiting time for a session is 9 days (minimum 0, maximum 38).

**Graph 2.**
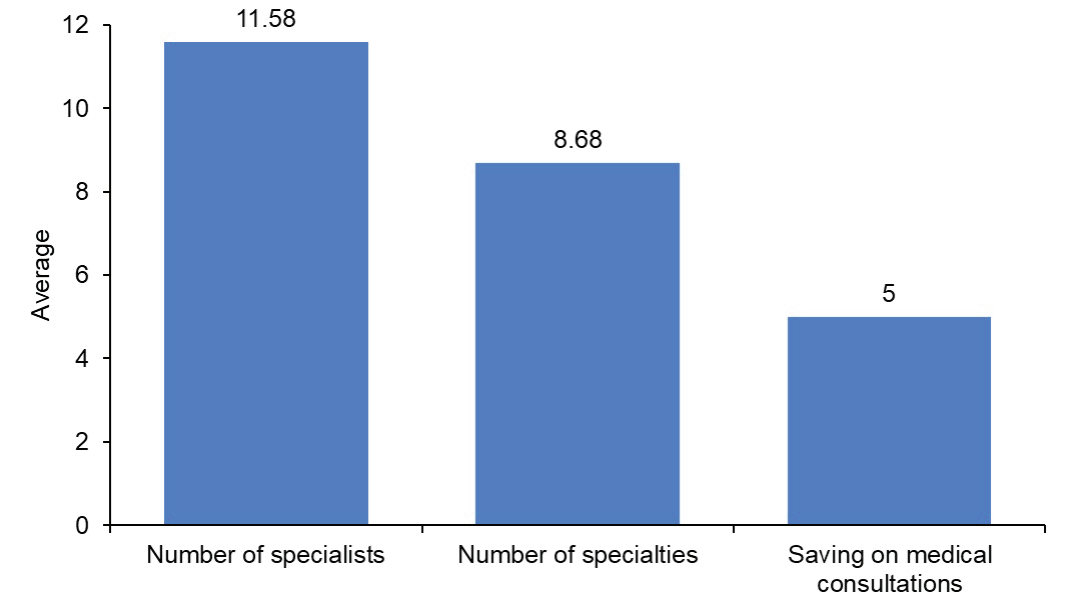
Average number of specialists, specialties and consultations saved by using multispecialty teams.

74.9% (*n* = 358) of the cases had a single session and 25.1% (*n* = 120) of the cases had recessions. The average number of medical specialties participating in the sessions was 8.68 (minimum 2, maximum 15), while the average number of specialists participating was 11.58 (minimum 3, maximum 19). Considering only the specific medical specialists for a single case who assisted in the sessions with the multispecialty team for decision making, an average cost savings of 5 consultations with medical specialists was generated (minimum 2, maximum 11), in addition to the savings in specialists from complementary and administrative units (Graph 2). With respect to Spearman's correlation, considering the number of specialties and consultations saved, a value of *p* = 0 <0.05 was found, which indicates that there is a significant relationship, i.e., a direct relationship: the greater the number of specialists, the more consultations saved; the correlation is regular, with a value of *p* = 0.595. Likewise, Spearman's correlation considering the number of doctors and consultations saved, obtained a value of *p* = 0 <0.05; therefore, there is a significant relationship, with a direct relationship, i.e., a direct relationship.

That is, the greater the number of physicians, the greater the number of consultations saved; with a high correlation (*p* value = 0.619).

**Graph 3.**
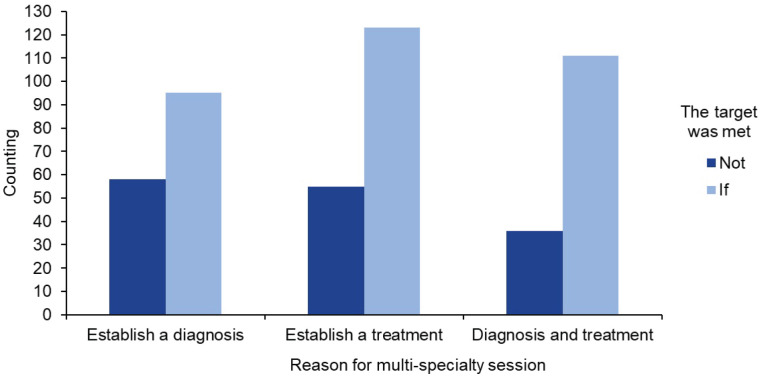
Count of reasons for multispecialty sessions and objective met for each reason.

The 68.8% of the sessions fulfilled the objective of the session. Graph 3 shows the count of the objectives fulfilled according to the type of reason for requesting the session: diagnosis, treatment or both. Chi-square obtained a value of *p* ≤ 0.05; therefore, there is a significant association between the reason for the session and the fulfillment of the session. Also, in the ANOVA result, a *p* ≤ 0.05 was obtained, which affirms that the reason for the session significantly influences the fulfillment of the objectives, which represents a valuable finding to optimise the multispecialty session model in the approach of oncology patients.

In 31.2% (*n* = 149) of the patients, it was not possible to establish whether the objective of the session was achieved, due to the lack of continuity in the follow-up of the case by the institution after the session. Of these patients, 38.93% (*n* = 58) could not establish a diagnosis due to lack of studies, 36.91% (*n* = 55) could not initiate treatment and 24.16% (*n* =36) could not establish both objectives (diagnosis and treatment) (Graph 3). The reasons identified included a lack of economic resources, and the decision to follow up in a public hospital or residence outside the metropolitan area of Guadalajara. However, they were given a minute with the conclusions and opinions of the multi-speciality team to be used wherever they considered necessary.

## Discussion

Studies showing the importance of integrating a team in the approach to patients refer to multidisciplinary teams, whose approach is more linked to the intervention of physicians specifically without integrating other professions [1, 3, 5, 7, 9–14]. Recent research indicates that these teams are not always truly multidisciplinary, i.e., they are a mixture of medical and paramedical disciplines, and that the medical profession (physicians and medical specialists) tends to dominate their interaction [6]. Similarly, in other articles, multi-specialisation is discussed with emphasis only on medical or health specialties [16]. In contrast, in this study, we speak of a multi-specialised team, in which all members, whether medical or other professional training, are highly trained personnel in the different areas involved to carry out individualised management and follow-up.

In this study, 478 cases were analysed in multi-specialised team sessions. The reason for the sessions had a constant variability, 37.2% of the sessions dealt with treatment, 32% with diagnosis and 30.8% with both. In those cases in which the reason was therapeutic, it was because they already had a previously confirmed diagnosis, while in the other two categories, they still had to corroborate it with different studies, evaluate a recurrence and reclassification or they were performed as a second opinion assessment. In 68.8% of the cases, the objective was achieved; while in 31.2% of the cases, there was no continuity; the main limiting factors were the lack of economic resources, the decision to have a follow-up in public institutions or being residents outside the metropolitan area of Guadalajara with travel difficulties. In spite of this, the corresponding minutes were given to the patients for their beneficial use in the institutions where they decided to continue with their treatment.

In the work of Takeda *et al* [14], they analysed 202 patients between April 2017 and June 2018, where they aimed to form a multidisciplinary committee to discuss cases of patients in whom it was difficult to formulate a diagnostic or therapeutic strategy; although they did not modify the TNM classification in any case, the diverse opinions led to correct the proposed strategies in 49 of them, that is, in 24.2%. On the other hand, in the study by Lumenta *et al* [13], the objective was the extended evaluation of multidisciplinary teams from the perspective of teamwork, in which 244 medical cases were discussed in 27 sessions, of which 228 (93%) of them reached an agreement for the recommendation of a treatment plan.

An outstanding aspect of the multi-specialised session model used at ION is the individualised classification in specific specialisation units for the patient's type of cancer; from the collection of files, it was found that 93.09% of them were part of some unit, while 6.91% were classified as non-specific; this last percentage is mainly due to the fact that the patients did not have continuity in their approach within the institute or did not have an accurate diagnosis because they had an unknown primary tumour with metastasis. With respect to the rest of the model, the study conducted by Takeda *et al* [14] in Japan uses a system similar to that of ION with respect to multidisciplinary virtual sessions in the area of lung cancer, which are held once a week on a continuous basis, lasting approximately 1 hour. Likewise, previous records are created about the patient's history, creating a Microsoft Word file. This team also liaises with other hospitals and external specialists to discuss the approach to difficult cases [14].

In contrast, at the institute in this study, such sessions are held twice a week, in addition to covering all types of cancer.

Similarly, in the article by Torrecillas-Torres *et al* [10] published in 2019, the selection of main specialties (surgery, pathology, radiology and endoscopy) is shared by a coordinator, and the previous preparation of a basic document with the patient's history for the selected areas. Likewise, the corresponding conclusions or recommendations are submitted for discussion with the aim of reaching a consensus [10]. However, in ION this basic document is given to the patient for free use with prior explanation of its pathophysiology and the approach indicated by the multi-specialised team. In contrast, the study conducted by Barrera-Franco *et al* [9] on the multidisciplinary approach to head and neck cancer in different public institutions in Mexico, shares with ION the prior socioeconomic, diagnostic and therapeutic investigation of the patient, to then carry out the sessions as a team; likewise, these institutions adopt continuous communication with hospitals and external specialists. However, it is mentioned that their sessions are not continuous and depend on the degree of complexity of the cases, with weekly or monthly frequency [9].

In terms of the number of sessions, 74.9% of cases required only one session, while the remaining 25.1% required 2 or more. This arises from the expectation of laboratory, cabinet or biopsy results, and therapies such as neoadjuvant chemotherapy, whose efficacy should be evaluated before proceeding with surgical management; in addition, in patients with recurrences to evaluate alternative approaches. The average waiting time from the consultation in which the multi-specialised virtual sessions are suggested currently takes place in a total of 9 days, due to the fact that each case requires an individualised approach, which includes the collection of data for the opinion of the integral approach and waiting for the results of laboratory studies, cabinet or biopsies; likewise, previously scheduled cases are considered, so there may be a delay in the generation of new sessions. However, if necessary, priority may be given to urgent cases due to the nature and severity of the same, with a delay of even less than 24 hours; these cases require immediate attention and rapid decision making by the medical team, to provide the best possible care to the patient, reviewing the most important details and establishing an appropriate plan of action.

Likewise, the units that require more time to schedule the session are those specialising in lung cancer, central nervous system tumours, lymphomas and leukemias, since their cases tend to be more complex and frequently metastasize. Gomez *et al* [17] carried out a study in which they evaluated the median diagnosis-treatment interval in patients with non-small cell lung cancer, which was 27 days; this delay was obtained in 57.4% of the patients, in association with the use of Positron Emission Tomography to evaluate its staging and dissemination. Another study conducted in Australia by Cook *et al* [18] aimed to evaluate whether primary treatment of head and neck cancer started within 56 days after the initial referral to the specialist, which is the pre-established time in their country. They found that the mean time was 45.5 days, and that 72% complied with the aforementioned guideline; the other 28% did not achieve the objective mainly because they were patients who underwent primary radiotherapy or who required insertion of a prophylactic gastrostomy tube [18].

The number of specialties present per session was 8.68 on average, while the number of specialists was 11.58. It is important to mention that more physicians usually participate in these meetings since more than one case is discussed per day, and despite having been convened for a specific case, all those who attend the session can participate and contribute in all cases. However, it was identified that the average number of medical specialists for each particular case was 5, ranging from 2 to 11, depending on the complexity of the case. Sometimes there is more than one professional per specialty, most of whom belong to the ION team, but may also be invited from other institutions. It should be noted that the specialists do not have an economic gain for participating in the multispecialty session to which they have been requested and that they voluntarily decided to participate and for the patient, these sessions do not imply an additional cost. When considering the number of medical specialists that help in the decision making in the approach of a patient, on average the patient saves 5 consultations in time and money, being an approximate saving of 5 hours and $6,000.00, but it should be noted that there were cases where the savings can reach 11 hours and $13,200.00 (in cases where 11 specialists are required). This does not take into account travel and scheduling time between appointments, as well as administrative equipment, nutrition and psychology. The study by Fleissig *et al* [7] mentions the reduction of costs with the implementation of multidisciplinary teams, but does not establish a specific amount; similarly, the article by Berardi *et al* [3] indicates that a group of specialists together can reduce the time in providing recommendations for the approach of medical cases, but do not comment on a defined duration [3]. In addition, there are facilities provided by the complementary ION team, in which financial and administrative advice is given, such as support in connection with insurance companies, scheduling of diagnostic studies, surgical procedures, hospitalisations, nursing services, psychological and nutritional support.

During the analysis of the units, it was found that the most frequently performed unit was that of breast tumours with 15%; on the other hand, the least frequent was that of robotic surgery with 0.8%. This implies that breast cancer has a higher frequency in relation to other types of tumours [15]. Furthermore, at the institute, this incidence is due to the fact that most patients with breast cancer are admitted to the oncologic surgery specialty, where sessions are held before a surgical procedure in which the opinion of medical oncology and radiation oncology is necessary to evaluate neoadjuvant treatment and the possibility of performing surgery.

Being a retrospective study, there were files with missing important data to be able to evaluate the model and because they did not meet the inclusion requirements, there were patients who were candidates for the study that were eliminated in the analysis process. It is essential that the various experts at the institute all contributed useful, relevant and strategic information to the ION operating system; in this way, proper implementation of data would have a significant impact on future research. The impossibility of following up patients due to socioeconomic factors and their place of residence after the multispecialty session, shows us part of the difficulties experienced by oncology patients and those who manage this type of patient; however, for this model of care, a strategy to consider would be to contact patients so that they can share whether the conclusions of the minutes were useful to them, respecting their confidentiality and their decision to continue their care in another institution. Similarly, another area of optimisation that could be carried out by the institute is the expansion of units, to eliminate the category of ‘other tumours’ and maintain individualised categorisation, since cases classified as others could not be considered in a specific unit during the study because the type of cancer did not fall within the current list of medical specialisation units currently on the platform.

## Conclusion

In conclusion, the study shows that the model of multispecialty virtual sessions for oncology patients carried out at ION provides significant benefits in terms of reduced waiting time, resource savings, access to specialised units and improvement of the patient's comprehensive approach. In addition, it is worth highlighting the importance of these teams and the definition of the term multispecialty by our institution, since it encompasses not only medical professionals, but also professionals from other areas such as administrative, financial, pharmaceutical, psychological, nutritional and nursing. With the results obtained regarding the fulfillment of the reason for the session, it shows that it is a successful and replicable model, where it is essential to have the coordination of the multispecialty team, to ensure the quality and completeness of the data, as well as the preparation and invitation prior to the sessions, the communication between the specialists, and with the patients and the implementation of the complementary units for the execution and fulfillment of the decisions taken.

The results underscore the need to promote and adopt this type of approach in medical practice to optimise clinical outcomes and improve patient management. Future prospective research should focus on overcoming logistical barriers and evaluating the long-term impact of these interventions in different settings and health systems.

## Conflicts of interest

The authors declare that they have no conflicts of interest with respect to the material discussed in the manuscript.

## Funding

The authors do not report the participation of any sponsor in the research that could have influenced the outcome of this work.

## Consent to use data

The National Cancer Institute has provided institutional informed consent for the use of data from patients' medical records for research purposes.

## Author contributions

Conceptualisation: LAG. Data Curation: KSB, ASN. Formal analysis: AGM. Research: KSB, ASN. Methodology: LAG.Project Management: AVT. Resources: LAG, MS, FA, GR, AD, JJ, KZ, SS, MQ. Software: AGM, KSB, ASN. Supervision: AVT, AGM. Validation: GR, JJ, SS, MS, AVT., AGM. Display: KSB., ASN. Writing – original draft: KSB, ASN. Writing – proofreading and editing: AVT, AGM.
